# EEG-timed neuromuscular stimulation shapes ipsilateral TMS-evoked motor responses in humans

**DOI:** 10.1016/j.cnp.2026.07.011

**Published:** 2026-07-21

**Authors:** Sebastian Schütz, Alireza Gharabaghi

**Affiliations:** a Institute for Neuromodulation and Neurotechnology, Department of Neurosurgery and Neurotechnology, University Hospital and University of Tübingen, 72076 Tübingen, Germany; b Center for Digital Health (CDH), 72076 Tübingen, Germany; c Cognitive Science Center (CSC), 72076 Tübingen, Germany; d Center for Bionic Intelligence Tübingen Stuttgart (BITS), 72076 Tübingen, Germany; e German Center for Mental Health (DZPG), 72076 Tübingen, Germany; f Max Planck-University of Toronto Centre for Neural Science & Technology (MPUTC), Germany

**Keywords:** EEG-timed stimulation, Neuromuscular electrical stimulation, Ipsilateral TMS-evoked motor responses, Sensorimotor beta activity, Transcranial magnetic stimulation, State-dependent neuromodulation

## Abstract

**Objective:**

Sensorimotor beta activity provides a real-time marker of motor-network state, but whether electroencephalography (EEG)-derived timing of peripheral stimulation can modulate ipsilateral motor output remains unclear. We tested whether EEG-timed neuromuscular electrical stimulation (NMES) alters ipsilateral transcranial magnetic stimulation (TMS)-evoked motor responses in humans.

**Methods:**

Twenty right-handed healthy participants completed four sessions of 24 Hz NMES applied to the left extensor digitorum communis. NMES train onset was triggered at four nominal timing conditions derived from the ongoing 24 Hz EEG component over the ipsilateral sensorimotor cortex. Ipsilateral motor output was assessed with TMS before and after each intervention. Peak-to-peak amplitudes of ipsilateral TMS-evoked motor responses were analyzed using a linear mixed-effects model.

**Results:**

EEG-timed NMES produced timing-dependent and muscle-specific changes in ipsilateral TMS-evoked motor response amplitudes. Flexor responses were suppressed across timing conditions, whereas extensor responses increased selectively in one EEG-derived timing condition. This response pattern dissociated generalized antagonist suppression from timing-specific facilitation of the stimulated extensor motor output.

**Conclusions:**

Ipsilateral TMS-evoked motor responses are sensitive to the temporal alignment between peripheral input and ongoing EEG-recorded ipsilateral sensorimotor activity.

**Significance:**

EEG-timed NMES provides a human neurophysiological framework for probing state-dependent sensorimotor coupling and may inform future closed-loop peripheral neuromodulation strategies.

## Highlights

• EEG-derived peripheral stimulation timing modulated ipsilateral TMS-evoked motor responses.
• Flexor responses were suppressed across all stimulation timing conditions.
• Extensor facilitation occurred selectively in one sensorimotor timing condition.

## 1 Introduction

Neuromuscular electrical stimulation (NMES) applies controlled electrical currents to peripheral nerves and muscles to evoke afferent and efferent activity (Carson and Buick, 2021). Originally developed for muscle strengthening and reeducation, NMES has become a key experimental tool to study the interaction between sensory feedback and motor control (Maffiuletti et al., 2018; Nussbaum et al., 2017). Through the concurrent activation of peripheral receptors and central motor circuits, NMES provides a means to probe sensorimotor coupling in the intact and injured nervous system (Bergquist et al., 2011; Carson and Buick, 2021), consistent with Hebbian principles of associativity (Hebb, 1949).

Peripherally, NMES elicits muscle contractions by direct activation of motor axons, leading to measurable force output and proprioceptive feedback (Maffiuletti, 2010). Centrally, it modulates cortical activity and corticospinal excitability (CSE), engaging both crossed and uncrossed motor pathways (Arendsen et al., 2021; Carson and Buick, 2021). While its effects on contralateral projections are well documented, the influence of NMES on ipsilateral motor output remains insufficiently characterized. Ipsilateral transcranial magnetic stimulation (TMS)-evoked motor responses in healthy individuals are known to arise from a combination of mechanisms, including uncrossed corticospinal projections, transcallosal interactions, reticulospinal pathways, and spinal commissural circuits (Alagona et al., 2001; Schwerin et al., 2011; Zaaimi et al., 2012). Because these mechanisms cannot be separated with noninvasive TMS and surface electromyography (EMG), we use the term ipsilateral TMS-evoked motor responses to describe the measured output without assigning it to a specific anatomical pathway. The ipsilateral motor system contributes to bilateral coordination, postural control, and recovery from corticospinal injury (Wilkins et al., 2020), yet its sensitivity to the timing of peripheral input remains insufficiently understood.

State-dependent stimulation approaches provide the conceptual basis for timing peripheral input to ongoing sensorimotor activity. Previous work has shown that coupling peripheral input with endogenous brain states enhances corticospinal responses. EMG-triggered NMES, motor imagery, and brain-computer interface (BCI) systems exploit ongoing motor-related activity to synchronize stimulation with central commands, thereby potentiating excitability through associative mechanisms (McGie et al., 2015; Monte-Silva et al., 2019; Mrachacz-Kersting et al., 2016; Mrachacz-Kersting et al., 2012; Mrachacz-Kersting et al., 2019; Royter and Gharabaghi, 2016). Such BCI-controlled NMES approaches have demonstrated promise in facilitating CSE (McGie et al., 2015) and promoting post-stroke motor recovery (Biasiucci et al., 2018; Cervera et al., 2018; Mane et al., 2020). However, their efficacy depends on reliable detection of motor-related activity, which can be limited in individuals with impaired voluntary activation. In individuals lacking sufficient voluntary muscle activation, the combination of NMES with antigravity assistance may be necessary (Grimm and Gharabaghi, 2016; Grimm et al., 2016). Motor imagery alone can increase CSE and modulate spinal circuitry (Chong and Stinear, 2017; Guggenberger et al., 2020a; Roosink and Zijdewind, 2010; Suzuki et al., 2023), though it is cognitively demanding (Guggenberger et al., 2020b), with some individuals lacking proficiency in it (Ahn et al., 2013). Hybrid paradigms combining electroencephalography (EEG) and EMG improve control reliability (Grimm and Gharabaghi, 2016). Respiration also influences motor excitability and cortical rhythms (Kluger and Gross, 2020; Li and Rymer, 2011; Ozaki and Kurata, 2015), suggesting that intrinsic physiological rhythms modulate sensorimotor communication. These lines of work converge on the principle that stimulation effects can depend on the physiological context in which afferent input is delivered, motivating EEG-based timing strategies that do not require voluntary motor output.

Neural oscillations, particularly in the beta band (13–30 Hz), have been implicated in coordinating sensory feedback, motor output, and the maintenance of motor-network state. The instantaneous phase of ongoing oscillatory activity can influence neuronal responsiveness to incoming input (Bonnefond et al., 2017; Chance et al., 2002; Salinas and Thier, 2000), and both the power and phase of sensorimotor beta activity have been linked to corticospinal excitability during rest and movement (Keute et al., 2023; Khademi et al., 2018, Khademi et al., 2019; Khademi et al., 2023; Naros et al., 2020). Brain-state-dependent stimulation studies have shown that aligning external input with specific endogenous activity states enhances CSE and recruits additional corticospinal pathways (Kraus et al., 2016; Kraus et al., 2018). These findings support the concept that motor responses to peripheral or cortical stimulation can depend on the ongoing EEG-derived activity state of the sensorimotor system. In the present experiment, sensorimotor state refers specifically to the EEG-recorded sensorimotor activity used for online timing of NMES train onset. Accordingly, the relevant “state” is operationalized as the estimated 24 Hz EEG component over the ipsilateral sensorimotor cortex.

However, beta activity is not a stationary oscillatory process. Recent work emphasizes that sensorimotor beta activity often occurs as transient bursts rather than as a continuous rhythm (Little et al., 2019; Lundqvist et al., 2024). This burst-like organization changes the interpretation of stimulation timed to beta-band activity: a beta peak or trough may not represent a fixed physiological phase with invariant neuronal meaning, but may instead index a broader brain state, such as burst emergence, burst maximum, burst decay, altered network gain, or transient stabilization of the motor system. Sensorimotor beta activity has also been linked to corticomuscular coupling and coherent communication between motor cortex and muscles (Liu et al., 2019; Peng et al., 2024). Thus, stimulation timed to the estimated 24 Hz EEG component may probe state-dependent sensorimotor coupling without requiring the assumption that the oscillatory phase itself is the sole causal variable.

Unilateral NMES can also alter excitability in the contralateral hemisphere (Carson and Buick, 2021) and modulate tone in non-stimulated antagonistic muscles (Li and Rymer, 2011; Suchetha et al., 2017). Such bilateral effects imply interhemispheric and ipsilateral contributions to sensorimotor regulation, possibly mediated by transcallosal or reticulospinal pathways (Asboth et al., 2018; Dewald et al., 1995; Mukherjee and Chakravarty, 2010; Schwerin et al., 2011; Zaaimi et al., 2012), with abnormal cortico-reticulospinal recruitment potentially contributing to spasticity and flexion synergies in stroke patients (Dewald and Beer, 2001). NMES has been shown to reduce spasticity following stroke (Stein et al., 2015). Testing whether NMES can modulate ipsilateral motor output when timed to ongoing EEG-recorded ipsilateral sensorimotor activity could therefore clarify how peripheral input interacts with intrinsic motor-network states.

The present study investigated whether NMES delivered at different time points relative to the ongoing 24 Hz EEG component modulates ipsilateral motor output. NMES was applied to the left extensor digitorum communis muscle and timed according to four nominal timing conditions derived from the ongoing 24 Hz EEG component over the ipsilateral sensorimotor cortex. The four timing conditions corresponded to quarter-cycle trigger timings of the estimated 24 Hz EEG component: rising flank, peak, falling flank, and trough. Because one 24 Hz cycle lasts about 40 ms, these nominal trigger offsets correspond to approximately 0, 10, 20, and 30 ms relative to the estimated rising flank. These values refer to EEG-derived NMES train-onset timing, not to the estimated cortical arrival phase of the afferent volley. Ipsilateral motor output was assessed with TMS before and after each intervention. We examined whether this EEG-timed stimulation produces timing-dependent and muscle-specific changes in ipsilateral motor output. This approach tests whether ongoing EEG-derived 24 Hz sensorimotor activity can serve as a real-time marker for state-dependent sensorimotor coupling in the human motor system.

## 2 Methods

### 2.1 Participants

Twenty right-handed adults (14 female; mean age = 25.2 years, SD = 4.6) participated after providing written informed consent. The study was approved by the Ethics Committee of the Medical Faculty, University of Tübingen, and conducted in accordance with the Declaration of Helsinki. All participants were aged 18–45 years, reported no regular use of medical or recreational drugs, and had no history of neurological, neurosurgical, or psychiatric treatment or recent sleep deprivation. Right-handedness was verified using the Edinburgh Handedness Inventory (Oldfield, 1971). Participants abstained from caffeine on the day of testing and had no contraindications to transcranial magnetic stimulation (TMS) (Rossi et al., 2021). Right-handed participants were selected to standardize hemispheric dominance. Stimulation was applied to the non-dominant left hand to maximize detectability of ipsilateral motor responses from the dominant left hemisphere, as ipsilateral TMS-evoked motor responses are more reliably observed under these conditions (Ziemann and Hallett, 2001).

### 2.2 Experimental procedure

**Fig. 1:**
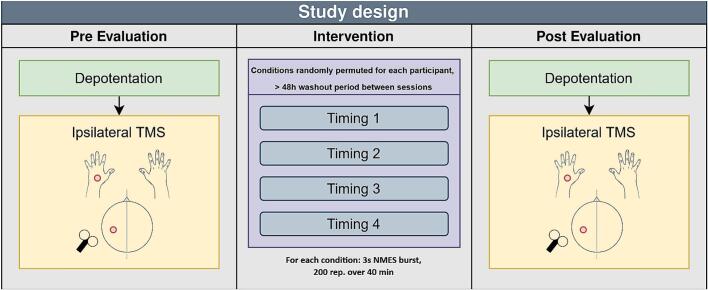
Experimental design. Each session included a pre-evaluation, NMES intervention, and post-evaluation phase. Ipsilateral motor output was assessed using transcranial magnetic stimulation (TMS) before and after the intervention. A depotentiation task preceded each TMS block to test whether intervention effects persisted beyond transient potentiation. This task involved 20 10 s contractions of the left forearm at 30% of maximal voluntary contraction using visual feedback. Ipsilateral TMS assessments used 120 pulses before and 120 pulses after the NMES intervention at 100% of the maximum stimulator output. Four EEG-derived NMES timing conditions were applied in randomized order, with one timing condition tested per session. Each session comprised 200 NMES epochs, and each epoch consisted of a 3 s NMES train delivered at 24 Hz. Sessions were separated by at least 48 h to reduce carryover effects.

Each participant completed four sessions (Fig. 1), separated by at least 48 h. A session was defined as one experimental visit testing one EEG-derived timing condition. Each session followed the same sequence: preparation, hotspot localization and resting motor threshold determination, pre-intervention TMS assessment, EEG-timed NMES intervention, and post-intervention TMS assessment. The order of the four timing conditions was randomized across participants.

EEG-timed NMES was applied to the left extensor digitorum communis (EDC) through Neuroline 720 surface electrodes connected to an STG 4008 stimulator (Multi Channel Systems GmbH). Each pulse was biphasic, comprising a 400 μs positive phase and a 400 μs negative phase, and was delivered at 24 Hz without ramping. Stimulation intensity was individually adjusted to the lowest level that produced clear and reproducible wrist and finger extension without visible flexor activation. Intensities ranged from 5 to 16 mA (mean ± SD = 9.2 ± 2.0 mA) and produced consistent activation without discomfort.

Participants were seated comfortably with the left forearm stabilized. During TMS assessments, the wrist and fingers were kept relaxed, and participants were instructed not to assist any response voluntarily. During NMES, the hand was positioned so that the stimulation-evoked EDC contraction raised the relaxed hand from a hanging position to approximately horizontal. No voluntary contraction of the target muscle was required during TMS or NMES. Pre-stimulus EMG activity was monitored and quantified to control for background muscle activation.

Ipsilateral motor output was assessed using TMS before and after each NMES intervention. To identify robust post-intervention effects, participants performed a depotentiation task immediately before the post-intervention TMS assessment (Goldsworthy et al., 2016; Todd et al., 2009; Touge et al., 2001). The same task was also conducted prior to the pre-intervention assessment to maintain procedural symmetry. During the depotentiation task, participants contracted the left extensor digitorum communis (EDC) at 30% of maximal voluntary contraction (MVC) for 10 s, using visual feedback on a computer screen. The task was repeated 20 times per session, preceding each TMS block. Electromyography (EMG) was recorded using Neuroline 720 (Ambu GmbH) surface electrodes in a belly-belly montage over forearm muscles: extensors (extensor digitorum communis [EDC], extensor carpi radialis [ECR]) and flexors (flexor digitorum superficialis [FDS], flexor carpi radialis [FCR]). Signals were amplified with an eego mylab amplifier (ANT Neuro, Germany).

Terminology was used as follows. A “timing condition” refers to one of the four EEG-derived trigger timings. The “intervention” refers to the 40-min NMES period within a session. A “stimulation epoch” or “NMES trial” refers to one 3 s NMES train. Because stimulation was delivered at 24 Hz, each 3 s epoch contained 72 electrical stimuli. Each session included 10 runs of 20 NMES epochs, corresponding to 200 epochs per session. A “TMS trial” refers to one TMS pulse during the pre- or post-intervention assessment block.

### 2.3 Assessment of ipsilateral motor output

TMS was delivered with a MagPro X100/R30 stimulator (MagVenture GmbH) using an MCF-B70 Butterfly coil. Neuronavigation (Localite GmbH) guided coil placement over the left primary motor cortex throughout the experiment to maintain reproducible coil position and orientation. Biphasic stimulation was applied at 45° to the longitudinal fissure in a posterior-anterior orientation. The coil orientation was optimized for contralateral hotspot identification. Approximately 40 pulses were used to locate the hotspot during each session. The hotspot was first determined for the contralateral right EDC. Stimulation began at 50% of maximal stimulator output (MSO) and was increased in 5% increments until reliable contralateral motor responses appeared. The three sites producing the largest peak-to-peak amplitudes were identified, each stimulated three additional times. The site with the highest average amplitude was defined as the hotspot. The resting motor threshold (RMT) was determined using the relative frequency method, defined as the lowest intensity eliciting contralateral motor responses >50 μV in 5 of 10 trials (Groppa et al., 2012).

Contralateral RMT was assessed to standardize hotspot identification, document individual corticospinal excitability, and confirm that the selected stimulation site was a conventional EDC motor hotspot. It was not used to scale the intensity for ipsilateral response assessment. Ipsilateral TMS-evoked motor responses are typically small and inconsistently elicited at conventional suprathreshold intensities; therefore, ipsilateral responses were assessed at 100% MSO, consistent with previous work on ipsilateral responses (Alagona et al., 2001).

Ipsilateral TMS-evoked motor responses were then recorded from the left EDC, ECR, FDS, and FCR by stimulating the same left-hemispheric hotspot at 100% MSO. Each session included 240 ipsilateral TMS assessment trials in total: 120 before the NMES intervention and 120 after the NMES intervention. EMG was recorded simultaneously from all four muscles during each TMS trial. Responses were measured as peak-to-peak voltages (Vpp) within 15–80 ms post-stimulus. Background EMG activity was quantified in a 15–80 ms pre-stimulus window. For each trial, the standard deviation (SD) of the rectified EMG signal within this interval was computed, and TMS-evoked motor responses were accepted only if their peak-to-peak amplitude exceeded 2 SD of baseline activity, thereby minimizing contamination by pre-activation.

The spatial precision of TMS was controlled by neuronavigation and reproducible coil placement, but stimulation at the EDC hotspot was not interpreted as selective activation of a single muscle representation. Because forearm muscle representations in primary motor cortex are adjacent and partly overlapping, TMS at the EDC hotspot may also recruit neighboring representations. This limitation was addressed by simultaneous EMG recordings from extensor and flexor muscles and by analyzing within-session pre-to-post changes under identical TMS parameters.

### 2.4 Intervention with neuromuscular electrical stimulation.

The four interventions differed in the timing of NMES train onset relative to ongoing ipsilateral sensorimotor 24 Hz activity (Fig. 1).

**Fig. 2:**
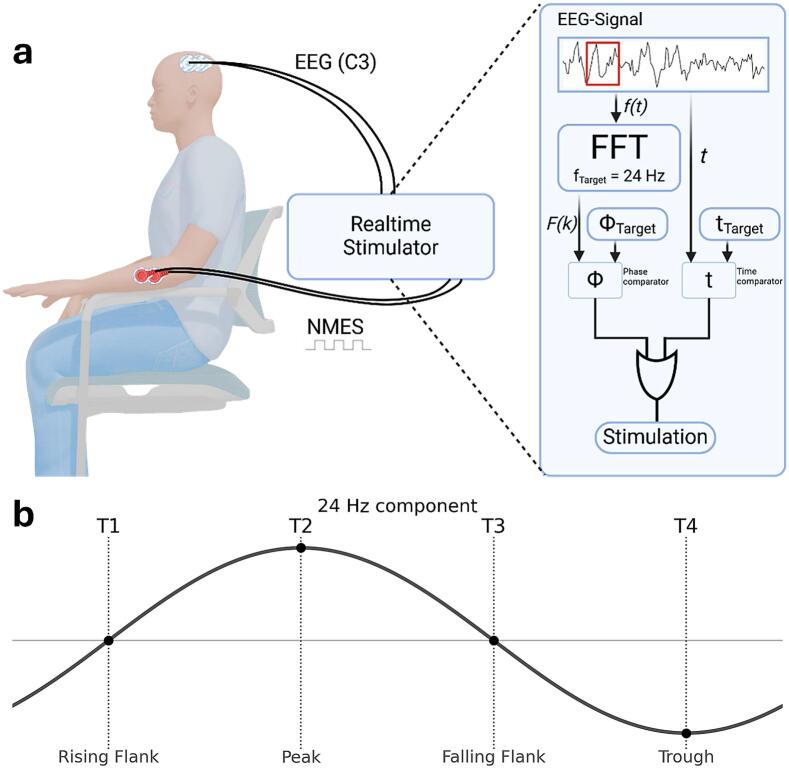
EEG-timed NMES. A. NMES was delivered to the left extensor digitorum communis muscle at 24 Hz. NMES train onset was timed according to the ongoing 24 Hz EEG component recorded over the ipsilateral sensorimotor cortex at C3. Real-time timing estimation was performed using fast Fourier transform-based analysis of the EEG signal in a 500 ms sliding window with zero-padding at 2 Hz resolution. B. Timing 1 to Timing 4 corresponded to the rising flank, peak, falling flank, and trough of the estimated EEG cycle, respectively. For a 24 Hz component, one cycle lasts about 40 ms; the nominal trigger offsets therefore correspond to approximately 0, 10, 20, and 30 ms relative to the estimated rising flank. These labels refer to EEG-derived triggering time points of NMES train onset, not to the phase at which the afferent volley reached the cortex, which was not estimated. Only the first pulse of each NMES train was EEG-timed; subsequent pulses followed the fixed 24 Hz stimulation rhythm. Stimulation intensity was individually adjusted to elicit EDC contraction, raising the relaxed hand from a hanging to a horizontal position.

During each session, NMES train onset was timed according to one of four nominal timing conditions derived from the ongoing 24 Hz EEG component at C3 (Fig. 2). Timing 1 to Timing 4 corresponded to the rising flank, peak, falling flank, and trough of the estimated EEG cycle, respectively. For a 24 Hz component, the cycle duration is about 40 ms; the four nominal trigger offsets therefore correspond to approximately 0, 10, 20, and 30 ms relative to the estimated rising flank. These labels refer to the EEG-derived triggering time points of NMES train onset, not to the phase at which the afferent volley reached the cortex, which was not estimated. Accordingly, the conditions are described as timing conditions rather than phase conditions throughout the manuscript. Only the first pulse of each stimulation train was EEG-timed. Subsequent pulses followed the fixed 24 Hz stimulation rhythm. Each session included approximately 200 stimulation epochs (40 min total), consisting of 3 s bursts of NMES per epoch. The pre-intervention TMS assessment was performed before the NMES intervention, and the post-intervention TMS assessment was performed after the NMES intervention.

Stimulation was controlled by an integrated measurement and stimulation device (IMSD, Loop-IT, NeuroConn, Ilmenau, Germany), which recorded EEG over the left sensorimotor area via a bipolar two-electrode montage 1 cm anterior and posterior to C3 (Guggenberger et al., 2023). EEG was analyzed online using a 500 ms sliding window with fast Fourier transform (FFT) and zero-padding at 2 Hz resolution. Triggering was based on the estimated 24 Hz EEG component; activity in other frequency bands was not used as a trigger criterion. The chosen window length reflects the inherent trade-off between frequency resolution and temporal responsiveness in real-time timing estimation: longer windows improve spectral precision but introduce latency that undermines closed-loop applicability. NMES train onset was triggered when the online EEG estimate matched the predefined timing condition. Subsequent pulses followed the fixed 24 Hz stimulation rhythm. This procedure aligns with previous work (e.g. Faller et al. (2022); George et al. (2023)) by restricting EEG-derived timing estimation to the first pulse of the stimulation train, which helps to avoid EEG-amplifier saturation.

To maximize stimulation specificity to the extensor compartment, electrode size and inter-electrode distance were kept small to increase current density and reduce current spread (Gomez-Tames et al., 2012). Functional verification of selective hand opening provided an additional control to ensure predominant activation of the extensor group.

Controlled slow breathing was implemented to stabilize physiological state and optimize EEG timing detection. Each breathing cycle lasted 10 s (3.3 s inspiration, 3.3 s expiration, 3.3 s rest). NMES was applied during expiration, 0.3 s after its onset, when corticospinal coherence is enhanced (Kluger and Gross, 2020).

A respiration belt (Go Direct, Vernier, Beaverton, Oregon) detected chest motion, and a closed-loop algorithm triggered a 3 s NMES train during expiration. Each session comprised 10 runs of 20 NMES epochs (200 total). Participants followed a visually guided breathing pattern supported by auditory cues. This approach aligned stimulation timing with respiratory phase, shown to modulate CSE (Li and Rymer, 2011; Ozaki and Kurata, 2015).

### 2.5 Data processing and statistical analysis

EMG data were bandpass-filtered (5–100 Hz) and notch-filtered at 50 Hz and harmonics. TMS-evoked motor responses were extracted as peak-to-peak amplitudes 15–80 ms post-stimulation. Background EMG was quantified for each TMS trial in a pre-stimulus window from 80 to 15 ms before the TMS pulse. The standard deviation of the rectified EMG signal within this window was used as an estimate of trial-specific baseline activity. Ipsilateral TMS-evoked motor responses were accepted only when the post-stimulus peak-to-peak amplitude exceeded two standard deviations of the corresponding pre-stimulus background EMG. This criterion reduced the likelihood that small post-stimulus deflections were classified as responses when they were not distinguishable from ongoing background activity. Outliers (<50 μV or > 3000 μV) were excluded. To normalize the non-Gaussian distribution (Capaday, 2021), amplitudes were log-transformed prior to analysis. Timing-dependent ipsilateral motor output modulation was assessed using a linear mixed-effects model with Time, Muscle, Timing, and their full factorial interaction as fixed effects:

$$\log\left(\mathrm{MEP}\right)∼\mathrm{Time}*\mathrm{Muscle}*\mathrm{Timing}+\left(1|\mathrm{Subject}/\mathrm{Muscle}\right)$$

Fixed effects were Time (pre vs post), Muscle group (EDC/ECR vs FDS/FCR), Timing (Timing 1–4) and their full factorial interaction. Subject was modeled as a random intercept, with Muscle nested within Subject. Pairwise contrasts were computed using the emmeans package (Lenth, 2022) to evaluate pre-to-post changes within each muscle group and timing condition, and to compare pre-to-post changes between timing conditions where appropriate. Bonferroni correction was applied to the post-hoc pairwise contrasts. Statistical inference was based on Wald z-tests with asymptotic degrees of freedom. All tests were two-tailed with an alpha level of 0.05. Analysis included *n* = 20 participants (total of 22,528 TMS-evoked motor responses). For clarity, results are presented as model estimates ± standard errors (SE), accompanied by z-ratios and *p*-values from the mixed-effects contrasts. To provide a standardized measure of effect magnitude, we calculated an approximate Cohen's d for the pre vs post contrasts of the estimated marginal means from the linear mixed-effects model. This was done by dividing the contrast estimate, the difference between estimated marginal means, by the residual standard deviation of the model. This approach approximates the traditional Cohen's d metric used in independent-samples designs, while acknowledging that it does not fully account for the variance attributable to nested random effects, such as subjects and muscles (Westfall et al., 2014). As such, the resulting d-values provide a practical, interpretable index of effect size but should be interpreted with caution. Model estimates and effect-size calculations are based on the log(response amplitude) scale; negative estimates correspond to proportional reductions in response amplitude. For visualization, raw response amplitudes are displayed on a logarithmic y-axis.

## 3 Results

EEG-timed NMES induced distinct timing- and muscle-specific changes in ipsilateral TMS-evoked motor responses.

### 3.1 Hand flexors

**Fig. 3:**
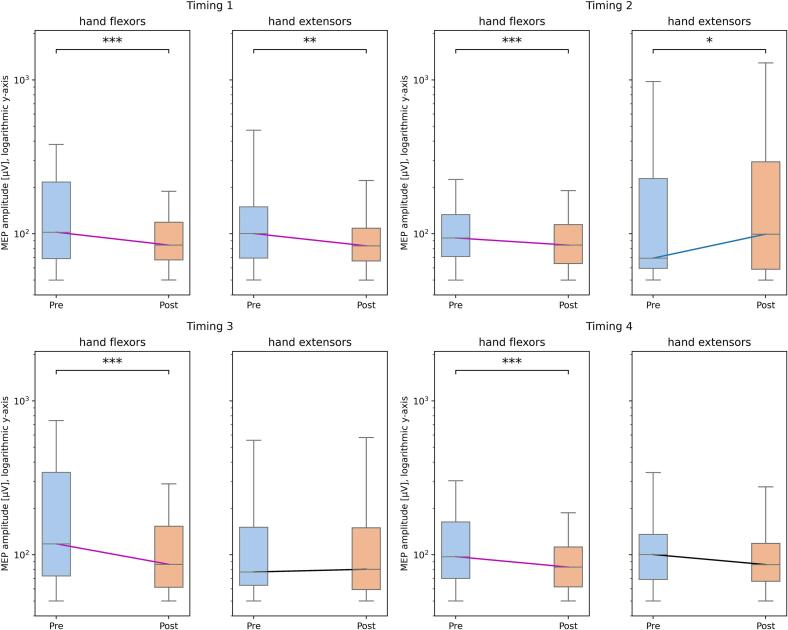
Timing-dependent changes in ipsilateral motor output. Boxplots show raw ipsilateral TMS-evoked motor response amplitudes for the four EEG-derived timing conditions, displayed on a logarithmic y-axis. Timing 1 to Timing 4 refer to NMES train-onset timing derived from the ongoing 24 Hz EEG component. Trend lines depict median pre-to-post changes in motor output: magenta indicates a decrease, blue indicates an increase, and black indicates no significant change. Statistical inference was based on log-transformed response amplitudes analyzed with a linear mixed-effects model, followed by Bonferroni-corrected post-hoc contrasts. Significance levels are denoted as * *p* < 0.05, ** *p* < 0.01, and *** *p* < 0.001. EEG-timed NMES produced a timing- and muscle-specific response pattern, with selective extensor facilitation in Timing 2 and generalized flexor suppression across timing conditions.

Across all four timing conditions, ipsilateral TMS-evoked motor response amplitudes of the hand flexors decreased significantly following NMES (Fig. 3):

Bonferroni-adjusted *p* < 0.0001 for all timing conditions; Timing 1: estimate = −0.0395, SE = 0.00435, z = −9.084, p < 0.0001, d = −0.33; Timing 2: estimate = −0.03305, SE = 0.00424, z = −7.798, p < 0.0001, d = −0.27; Timing 3: estimate = −0.07733, SE = 0.00434, z = −17.800, p < 0.0001, d = −0.64; Timing 4: estimate = −0.04676, SE = 0.00488, z = −10.427, p < 0.0001, d = −0.39. This consistent reduction indicates generalized suppression of ipsilateral motor output in antagonist muscles of the stimulated extensors.

### 3.2 Hand extensors

Hand extensors showed timing-specific modulation. Extensor TMS-evoked motor response amplitude increased significantly in Timing 2 (estimate = 0.01507, SE = 0.00533, z = 2.829, *p* = 0.0047, d = 0.12; Fig. 3). No significant pre-to-post modulation occurred in Timing 3 (estimate = −0.00689, SE = 0.00434, z = −1.587, *p* = 0.1125, d = −0.06) or Timing 4 (estimate = 0.00512, SE = 0.00410, z = 1.250, *p* = 0.2114, d = 0.04). In contrast, Timing 1 produced a significant decrease in extensor TMS-evoked motor response amplitude (estimate = −0.01722, SE = 0.00488, z = −3.527, *p* = 0.0004, d = −0.14).

### 3.3 Timing-specific muscle effects

Interaction-derived post-hoc contrasts of pre-to-post changes showed that extensor TMS-evoked motor response modulation in Timing 2 differed significantly from Timing 1 (*p* < 0.01) and Timing 3 (*p* < 0.05), indicating timing-specific modulation of extensor output.

Fig. 3 presents boxplots of raw TMS-evoked motor response amplitudes across EEG-derived timing conditions and muscle groups. The boxplots depict median response amplitudes and interquartile ranges across conditions. Standard significance markers (*, **, ***) indicate statistically reliable pre-to-post differences and timing-specific contrasts.

To provide the absolute amplitude scale underlying the logarithmically transformed analyses, non-transformed peak-to-peak response amplitudes are reported in Table 1. Values are presented as median [interquartile range] because response amplitudes showed skewed distributions.

**Table 1:** Raw detected ipsilateral TMS-evoked motor response amplitudes.

Condition	Muscle Group	Pre MRA, μV Mdn [Q1, Q3]	Post MRA, μV Mdn [Q1, Q3]
Timing 1	Extensors	100.336 [69.509, 149.674]	83.482 [66.655, 108.742]
	Flexors	102.399 [68.980, 217.169]	84.457 [67.655, 118.939]
Timing 2	Extensors	69.433 [59.643, 228.946]	99.475 [58.866, 293.684]
	Flexors	93.933 [71.231, 133.259]	84.400 [64.063, 114.894]
Timing 3	Extensors	100.232 [69.140, 135.610]	86.216 [67.232, 118.452]
	Flexors	97.144 [70.146, 163.238]	83.038 [61.817, 112.201]
Timing 4	Extensors	77.176 [63.297, 150.920]	80.410 [59.443, 149.770]
	Flexors	117.647 [72.754, 343.020]	86.637 [61.450, 153.289]

Values are shown for each condition and time point as median and the 25th and 75th percentiles. Motor response amplitudes represent peak-to-peak EMG amplitudes of detected ipsilateral TMS-evoked motor responses in the predefined post-stimulus response window.

Pre-stimulus background EMG remained low across conditions and muscle groups. Background EMG values are reported in Table 2. These data provide a control for background activation and support the interpretation that the observed pre-to-post changes were not explained by systematic changes in pre-stimulus muscle activity.

**Table 2:** Pre-stimulus background EMG RMS amplitudes before and after the intervention.

Condition	Muscle Group	Pre bEMG RMS, μV Mdn [Q1, Q3]	Post bEMG RMS, μV Mdn [Q1, Q3]	Delta Post-Pre, μV Mdn [Q1, Q3]	Statistics
Timing 1	Extensors	18.32 [16.19, 34.14]	16.34 [9.36, 24.80]	−3.18 [−15.49, 0.01]	W = 43; *p* = 0.036; pFDR = 0.289
	Flexors	20.10 [5.30, 31.17]	21.78 [7.57, 29.28]	−2.00 [−2.92, 4.77]	W = 85; *p* = 0.709; pFDR = 0.782
Timing 2	Extensors	19.98 [17.19, 26.64]	20.41 [15.48, 41.48]	−1.70 [−6.23, 1.23]	W = 34; *p* = 0.455; pFDR = 0.606
	Flexors	29.04 [17.91, 35.31]	23.47 [17.19, 36.55]	−4.30 [−12.34, 1.74]	W = 48; *p* = 0.190; pFDR = 0.380
Timing 3	Extensors	22.05 [13.84, 44.50]	24.06 [15.86, 34.96]	−4.61 [−13.47, 1.09]	W = 52; *p* = 0.087; pFDR = 0.350
	Flexors	20.56 [10.75, 30.71]	19.51 [12.70, 23.05]	−1.71 [−9.81, 2.68]	W = 68; *p* = 0.293; pFDR = 0.470
Timing 4	Extensors	21.28 [14.48, 27.33]	20.46 [7.19, 26.61]	1.54 [−10.93, 7.43]	W = 70; *p* = 0.782; pFDR = 0.782
	Flexors	25.45 [14.78, 74.67]	22.12 [18.33, 48.77]	−4.05 [−24.29, 3.54]	W = 40; *p* = 0.159; pFDR = 0.380

bEMG = background electromyography; RMS = root mean square; Mdn = median; Q1 = 25th percentile; Q3 = 75th percentile; Δ = post-intervention minus pre-intervention difference. Background EMG RMS was calculated in the pre-stimulus window from −85 to −15 ms relative to the TMS stimulus. Flexors included FDS and FCR; extensors included EDC and ECR. *p* values are based on paired pre-post Wilcoxon signed-rank tests; pFDR values were corrected for multiple comparisons using the false discovery rate procedure.

**Fig. 4:**
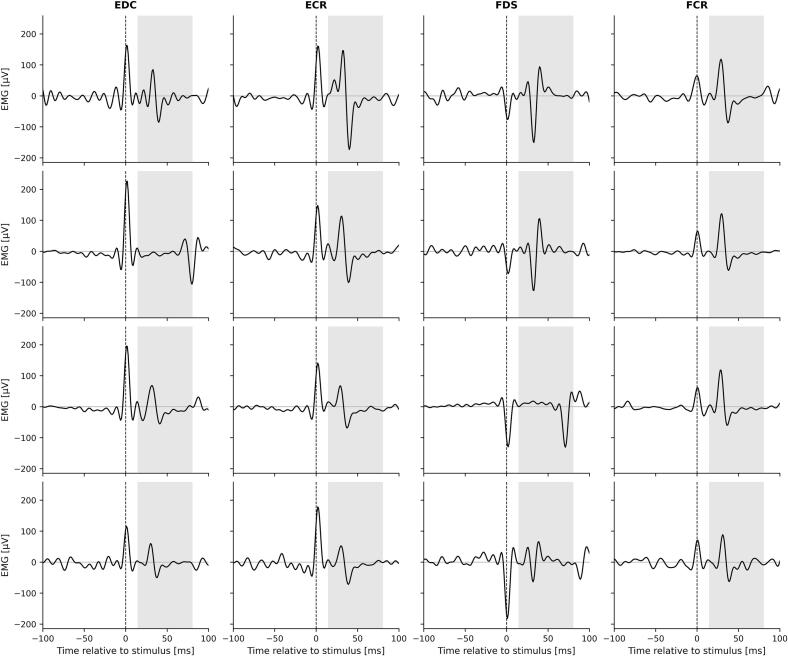
EMG recordings of ipsilateral TMS-evoked motor responses. Four exemplary trials of ipsilateral TMS-evoked motor responses from one representative participant and condition are shown for EDC, ECR, FDS, and FCR. TMS was delivered over the left motor cortex at 100% of maximum stimulator output. EMG data were band-pass filtered from 5 to 100 Hz and notch-filtered at 50 Hz and its harmonics. The gray shaded area marks the 15–80 ms post-stimulus window used for peak-to-peak amplitude detection. Note the trial-to-trial variability across muscles.

Representative raw EMG recordings are shown in Fig. 4 to illustrate the morphology and variability of the detected ipsilateral TMS-evoked motor responses. The examples display simultaneous recordings from EDC, ECR, FDS, and FCR in the predefined 15–80 ms post-stimulus response window used for peak-to-peak amplitude detection.

## 4 Discussion

The present study shows that NMES timed to ongoing EEG-derived 24 Hz over the ipsilateral sensorimotor cortex modulates ipsilateral motor output in a timing- and muscle-specific manner. NMES delivered to the left extensor digitorum communis produced a consistent suppression of ipsilateral flexor TMS-evoked motor response amplitudes across all timing conditions, whereas ipsilateral extensor TMS-evoked motor response amplitudes increased selectively in one timing condition. Effect sizes were consistently larger for flexor suppression than for extensor modulation, indicating a dominant antagonist-suppression effect. This muscle-specific asymmetry is consistent with evidence that sensory-input-dependent plasticity differs across human hand and forearm muscles, with intrinsic hand muscles and flexors showing greater plastic potential than extensors during paired associative stimulation (Foysal and Baker, 2019). Thus, the relatively small extensor facilitation observed here may reflect not only the timing dependence of EEG-timed NMES, but also a lower susceptibility of extensor output to sensory-driven plastic modulation. Together, these findings indicate that the effect of peripheral stimulation on ipsilateral motor output varied with the temporal relationship between NMES train onset and ongoing EEG-recorded ipsilateral sensorimotor activity. The results support the interpretation that EEG-derived timing can index motor-network states with different susceptibility to afferent input.

The extensor digitorum communis was selected as the target muscle because wrist and finger extension plays a critical role in functional hand opening, which is commonly impaired after stroke. Post-stroke upper limb impairment is frequently characterized by flexor-dominant synergies (Dewald and Beer, 2001), with weakness in finger extension being a major driver of impairment after stroke (Kamper et al., 2006). Even small gains in extensor activation can substantially improve grasp release and overall hand function (Lang et al., 2009), thereby enhancing independence in daily activities.

Because ipsilateral motor responses may arise from a combination of uncrossed corticospinal, transcallosal, subcortical, and spinal mechanisms, the present findings should be interpreted as modulation of ipsilateral motor output rather than modulation of a specific anatomical pathway. This terminology is important because noninvasive TMS and surface EMG cannot determine whether the observed ipsilateral TMS-evoked motor response changes were mediated by uncrossed corticospinal projections, transcallosal interactions, reticulospinal pathways, spinal commissural circuits, or their combination.

Although NMES-induced facilitation of contralateral excitability is well documented (Carson and Buick, 2021), modulation of ipsilateral motor output has received less attention. Previous studies using peripheral magnetic stimulation reported ipsilateral facilitation (Sato et al., 2018), but deeper field penetration and limited control over stimulation timing constrain mechanistic interpretation (Machetanz et al., 1994). The present study extends this work by showing that the effect of NMES on ipsilateral motor output varies across EEG-derived timing conditions, as indicated by interaction-derived post-hoc contrasts. This finding suggests that ipsilateral sensorimotor circuits are not only responsive to repetitive peripheral input, but also sensitive to the EEG-recorded sensorimotor activity state in which that input is delivered.

The present results should be interpreted in light of previous work linking beta activity to corticospinal excitability and state-dependent stimulation effects. Studies using beta-informed or phase-dependent stimulation have shown that corticospinal output can vary with the instantaneous state of sensorimotor oscillations (Keute et al., 2023; Khademi et al., 2018, Khademi et al., 2019; Khademi et al., 2023; Naros et al., 2020). Brain-state-dependent stimulation can also enhance CSE and recruit additional corticospinal pathways (Kraus et al., 2016; Kraus et al., 2018). However, the present study does not establish that a specific cortical beta phase was reached by the afferent volley. The timing labels used here refer to EEG-derived triggering time points of NMES train onset, not to verified cortical arrival phases. Therefore, the selective extensor facilitation observed in one specific condition should be interpreted as a timing-specific effect relative to the online EEG signal, not as direct evidence for causal stimulation of a specific beta phase.

This interpretation is consistent with the increasingly recognized nonstationary structure of beta activity. Sensorimotor beta activity is often expressed as transient bursts rather than as a sustained sinusoidal rhythm (Little et al., 2019; Lundqvist et al., 2024). In this framework, a timing condition derived from the estimated 24 Hz EEG component may not represent a fixed physiological phase with invariant neuronal meaning. Instead, it may index a broader motor-network state, such as beta-burst emergence, burst maximum, burst decay, transient stabilization of the motor system, altered sensorimotor gain, or changes in corticospinal and corticomuscular coupling. This view is also consistent with evidence that beta-band corticomuscular coherence reflects functional coupling between motor cortex and muscle activity (Liu et al., 2019; Peng et al., 2024). Thus, EEG-timed NMES may probe state-dependent sensorimotor coupling without requiring the assumption that beta phase itself is the sole causal variable.

The present response profile supports a cautious interpretation of the timing effect. Contralateral phase-dependent TMS studies have reported sinusoidal modulation of corticospinal excitability across the beta cycle (Keute et al., 2023; Khademi et al., 2018). In the present study, extensor facilitation was limited to one EEG-derived timing condition, whereas the remaining conditions showed either no significant modulation or reduced TMS-evoked motor response amplitudes. This selective pattern may reflect differences between contralateral corticospinal output and ipsilateral motor output, which likely depends on distributed interhemispheric, subcortical, and spinal circuitry. It may also reflect the fact that NMES train onset was timed to the online EEG signal, whereas the cortical arrival phase of the afferent volley was not estimated. Direct comparisons of ipsilateral and contralateral motor output within the same EEG-timed NMES framework will be required to determine whether these systems differ in their sensitivity to ongoing sensorimotor state.

The consistent reduction of flexor TMS-evoked motor response amplitudes across all timing conditions indicates that NMES engaged mechanisms affecting non-stimulated antagonist muscles. Similar effects have been reported for extensor stimulation reducing flexor tone or flexor excitability (Aimonetti and Nielsen, 2001; Li and Rymer, 2011; Suchetha et al., 2017; Sugawara et al., 2019). This pattern may reflect reciprocal inhibitory processes, spinal inhibitory circuits, or supraspinal sensorimotor interactions that coordinate flexor-extensor balance. The dissociation between generalized flexor suppression and selective extensor facilitation suggests that EEG-timed NMES did not produce a uniform global shift in motor excitability, but instead shaped ipsilateral motor output in a muscle-specific manner.

Importantly, the consistent suppression of flexor TMS-evoked motor responses suggests that NMES itself may exert a generalized inhibitory influence on non-stimulated antagonist muscles. Stimulation frequency is known to shape spinal and cortical responses, with repetitive peripheral stimulation recruiting presynaptic inhibition, reciprocal inhibitory circuits, and supraspinal gain-control mechanisms (Bergquist et al., 2011; Carson and Buick, 2021). Thus, the consistent flexor suppression observed across all timing conditions likely reflects a background effect of repetitive afferent input on ipsilateral motor output. Within this framework, the extensor facilitation observed in one condition may represent timing-specific modulation superimposed on a broader inhibitory background induced by the stimulation protocol.

These findings integrate with growing evidence that the ipsilateral motor system contributes actively to hand control and adaptation. Increases in ipsilateral corticospinal representations (Belardinelli et al., 2017; Khademi et al., 2022; Trunk et al., 2022) and reticulospinal integrity (Karbasforoushan et al., 2019) have been linked to altered motor control in stroke, while contralesional hyperexcitability correlates with severe impairment (Veldema et al., 2021). The present findings add a timing dimension to this literature by showing that ipsilateral motor output can be modulated by peripheral stimulation delivered relative to ongoing EEG-recorded sensorimotor activity. These findings support EEG-timed NMES as a framework for probing state-dependent sensorimotor coupling and for developing state-dependent peripheral stimulation strategies that exploit endogenous motor-network dynamics.

Because ipsilateral TMS-evoked motor responses can be small and difficult to distinguish from background EMG fluctuations, we added representative raw EMG traces and non-transformed response amplitudes to provide the absolute amplitude scale and response morphology. Pre-stimulus background EMG was quantified to control for background activation. The response-detection criterion was based on trial-specific baseline variability and was applied identically across conditions, making response detection more conservative in trials with higher background activity. These controls support the interpretation that the observed effects were not primarily driven by systematic changes in pre-stimulus muscle activation.

Several limitations should be considered. The study was performed in healthy right-handed participants, so generalizability to left-handed individuals or patients with corticospinal damage remains to be tested. Noninvasive measures cannot resolve the precise neuronal subpopulations or pathways mediating the observed effects, although invasive recordings could address this issue in future work (Shen et al., 2023). The analysis focused on immediate changes in TMS-evoked motor response amplitudes; the persistence of EEG-timed NMES effects after repeated interventions remains unknown. Controlled slow breathing was included to stabilize physiological state and support timing detection. However, additional control conditions using natural breathing or stimulation during alternative respiratory phases will be required to clarify the specific contribution of respiratory entrainment to the observed effects (Kluger and Gross, 2020; Ozaki and Kurata, 2015).

A related methodological limitation is the spatial precision of TMS. Neuronavigation and a figure-of-eight coil allowed reproducible stimulation of the same left-hemispheric hotspot, but focal TMS cannot selectively activate only the cortical representation of the EDC. The cortical representations of EDC, ECR, FDS, and FCR are close and partially overlapping. Therefore, TMS may have influenced neighboring representations and produced responses in multiple muscles. This does not invalidate the pre-to-post comparison, because the same TMS site, coil orientation, and intensity were used before and after each intervention; however, it limits anatomical specificity and supports our use of the broader term ipsilateral TMS-evoked motor responses.

An additional limitation concerns the high number of NMES pulses delivered per session, which is similar in volume to many paired associative stimulation paradigms. Although sessions were separated by at least 48 h, a washout interval commonly used in human plasticity studies (Stefan et al., 2000), the persistence of plasticity following EEG-timed peripheral stimulation has not been systematically characterized. Carry-over effects across sessions therefore cannot be excluded. This risk was mitigated by acquiring fresh baseline measurements at the beginning of each session and focusing the primary analyses on within-session pre-to-post changes. Future studies should explicitly examine the temporal stability, retention, and cumulative effects of repeated EEG-timed NMES across multiple days.

The present study was designed as a mechanistic investigation of timing-dependent modulation of ipsilateral motor output and did not include behavioral or kinematic endpoints. Consequently, the functional significance of the observed TMS-evoked motor response changes cannot be inferred directly. While TMS-evoked motor response amplitude is a well-established proxy for motor-system responsiveness, future studies should determine whether EEG-timed NMES translates into measurable improvements in motor performance, dexterity, force control, or flexor-extensor coordination. The depotentiation protocol involved a brief 30% MVC contraction prior to excitability assessment. Voluntary contraction is known to induce transient beta event-related desynchronization (Pfurtscheller and Lopes da Silva, 1999). However, the contraction period was short and applied symmetrically before both pre- and post-intervention assessments. Any transient modulation of beta power would therefore be expected to affect both assessments equivalently.

The study did not include a shuffled-timing or asynchronous stimulation control condition. Therefore, although the differential effects across timing conditions argue against a purely uniform effect of repetitive NMES, the specific contribution of EEG-derived timing cannot be isolated definitively. Similar state-dependent stimulation paradigms have reported corticospinal modulation without explicit asynchronous controls (Keute et al., 2023), but inclusion of a random-timing condition would strengthen causal inference. Future experiments directly comparing EEG-timed and non-EEG-timed NMES will be necessary to isolate the contribution of sensorimotor-state timing beyond general effects. Individual differences in beta dynamics, burst structure, attention, and fatigue may also contribute to variability in responsiveness and should be addressed in future work.

In conclusion, NMES timed to ongoing EEG-derived 24 Hz activity over the ipsilateral sensorimotor cortex revealed a timing-dependent and muscle-specific modulation of ipsilateral motor output. The observed pattern, generalized flexor suppression with selective extensor facilitation in one EEG-derived timing condition, suggests that the effect of peripheral stimulation depends on the ongoing sensorimotor state. These findings support EEG-timed NMES as a framework for probing state-dependent sensorimotor coupling and for developing closed-loop neuromodulation strategies that exploit endogenous motor-network dynamics.

## Declaration of generative AI and AI-assisted technologies in the writing process

The authors have read and approved the manuscript. It underwent thorough revisions by the authors. Additionally, the manuscript was refined for grammatical accuracy, sentence structure and wording using a convolutional neural network-based tool (DeepL) and an advanced language model (ChatGPT).

## Funding

This investigator-initiated study was supported by the German Federal Ministry of Research, Technology and Space (BMFTR) through the BEVARES (13GW0570) grant. The funding had no impact on the study design, on the collection, analysis and interpretation of data, on the writing of the report or on the decision to submit the article for publication.

## Declaration of competing interest

The authors declare the following financial interests/personal relationships which may be considered as potential competing interests: [AG was supported by research grants from the German Federal Ministry of Education and Research (BMBF), the European Union's Joint Program for Neurodegenerative Disease Research (EU-JPND), Medtronic, Abbott, and Boston Scientific, all of which were unrelated to this work].
